# Dengue envelope-based ‘four-in-one’ virus-like particles produced using *Pichia pastoris* induce enhancement-lacking, domain III-directed tetravalent neutralising antibodies in mice

**DOI:** 10.1038/s41598-018-26904-5

**Published:** 2018-06-05

**Authors:** Ravi Kant Rajpoot, Rahul Shukla, Upasana Arora, Sathyamangalam Swaminathan, Navin Khanna

**Affiliations:** 1 Recombinant Gene Products Group, Molecular Medicine Division, International Centre for Genetic Engineering & Biotechnology, New Delhi, India; 2 Translational Health Science & Technology Institute, NCR Biotech Science Cluster, Faridabad, India; 3 Department of Pediatrics, Division of Infectious Diseases, Emory University School of Medicine, Atlanta, GA USA

## Abstract

Dengue is a significant public health problem worldwide, caused by four antigenically distinct mosquito-borne dengue virus (DENV) serotypes. Antibodies to any given DENV serotype which can afford protection against that serotype tend to enhance infection by other DENV serotypes, by a phenomenon termed antibody-dependent enhancement (ADE). Antibodies to the viral pre-membrane (prM) protein have been implicated in ADE. We show that co-expression of the envelope protein of all four DENV serotypes, in the yeast *Pichia pastoris*, leads to their co-assembly, in the absence of prM, into tetravalent mosaic VLPs (T-mVLPs), which retain the serotype-specific antigenic integrity and immunogenicity of all four types of their monomeric precursors. Following a three-dose immunisation schedule, the T-mVLPs elicited EDIII-directed antibodies in mice which could neutralise all four DENV serotypes. Importantly, anti-T-mVLP antibodies did not augment sub-lethal DENV-2 infection of dengue-sensitive AG129 mice, based on multiple parameters. The ‘four-in-one’ tetravalent T-mVLPs possess multiple desirable features which may potentially contribute to safety (non-viral, prM-lacking and ADE potential-lacking), immunogenicity (induction of virus-neutralising antibodies), and low cost (single tetravalent immunogen produced using *P. pastoris*, an expression system known for its high productivity using simple inexpensive media). These results strongly warrant further exploration of this vaccine candidate.

## Introduction

Dengue is a mosquito-borne viral disease which threatens nearly half the world population^1^. Its prevalence has been reported in over a 100 countries around the world and its incidence is estimated to have increased ~30-fold in the past 5 decades^2^. Recent estimates indicate that there are annually ~390 million dengue infections around the world, of which ~25% result in clinical illness^3^. Dengue is caused by infection with any of four antigenically distinct dengue viruses (DENV-1, -2, -3 and -4), which belong to the genus *Flavivirus* of the family *Flaviviridae*^4,5^. Clinical illness caused by DENVs ranges from mild self-limiting dengue fever to severe, potentially fatal, dengue hemorrhagic fever and dengue shock syndrome^6^. Severe dengue disease is widely held to be the result of increased virus load arising from a phenomenon known as antibody-dependent enhancement (ADE), wherein antibodies to a particular DENV serotype, which can confer lifetime homotypic immunity to that serotype, bind and promote the uptake of a heterotypic DENV into Fcγ receptor (FcγR)-bearing cells and augment the severity of dengue illness^7^. Therefore, an effective dengue vaccine must be tetravalent, capable of inducing simultaneous immunity to all four DENVs, to avoid the risk of severe disease through ADE^8^. Recently, a tetravalent live-attenuated vaccine (LAV), Dengvaxia, has been licensed in several dengue-endemic countries^6,9^. However, this vaccine is only marginally effective against DENV-2^10,11^ and is suspected to sensitise naïve recipients to increased risk of hospitalisation at later times^12,13^. Two additional tetravalent LAVs, one being developed by the National Institutes of Health, USA^14^, and the other, by the drug company Takeda^15^, have entered phase III trials.

We have been exploring recombinant subunit vaccine alternatives based on *Pichia pastoris*-expressed DENV envelope antigens^16–20^ which possess inherent capacity to assemble into virus-like particles (VLPs). The envelope antigen, which is a ~500 amino acid (*aa*) residue-long glycoprotein, mediates critical events in viral entry such as host receptor recognition and membrane fusion, and is the major target of neutralising antibodies (nAbs)^4,5^. The N-terminal 80% of envelope, constituting the ectodomain (E), is organised into discrete domains known as envelope domains (ED) I, II and III^21^. Interestingly, the host receptor recognition site^22–24^ and serotype-specific potent neutralising epitopes^25,26^ map to EDIII. The prM protein which plays a role in virus maturation^4^ on the other hand carries cross-reactive epitopes that elicit non-nAbs, implicated in mediating ADE^27–29^.

Several studies have shown that prM and E can assemble together to form VLPs in the absence of viral genomic RNA^30–34^. However, in the light of the role of anti-prM antibodies in ADE, prM is an undesirable component of these VLPs. A serendipitous discovery we made a few years ago showed that the DENV-2 E ectodomain (referred to hereafter as ‘E2’), expressed in the absence of prM, in *P. pastoris*, possessed the inherent ability to self-assemble into highly immunogenic VLPs^16^. Subsequently, we found this to be true for *P. pastoris*-expressed E ectodomains corresponding to DENV-1^18^, DENV-3^17^ and DENV-4^19^, referred to hereafter as E1, E3 and E4, respectively. These VLPs were found to retain the antigenic integrity of their E monomers largely intact, based on probing with a battery of previously reported human and murine monoclonal antibodies (mAbs)^26,35–39^. Of note, all these VLPs elicited predominantly homotypic nAbs specific to their cognate serotypes. E2 VLPs conferred protection to AG129 mice against lethal DENV-2 challenge^16^ and E3 VLPs were found to lack significant ADE potential^17^. Further, nAbs elicited by these VLPs appeared to be directed almost exclusively to the C-terminally located EDIII, indicating that the VLPs actually serve as efficient EDIII display platforms. It is noteworthy that the receptor-binding^22–24^ and serotype-specific nAb-inducing epitopes^25,26^ of the E protein map to EDIII. Taken together, the inherent safety (lack of prM), immunogenicity (the capacity to elicit virus-neutralising anti-EDIII antibodies), and the low cost of production (*P. pastoris* expression system) make the E-based VLPs ideally suited to development of tetravalent dengue vaccine candidates.

While it is possible to physically mix these 4 monovalent E VLPs into a single tetravalent formulation, we were interested to explore if it would be possible to co-express all four E VLPs to create ‘four-in-one’ mosaic VLPs (mVLPs). Such an approach would make the candidate vaccine more cost-effective by circumventing the need to express and purify four monovalent E VLPs. We first ascertained the feasibility of co-expressing two different Es and their ability to co-assemble into bivalent mVLPs^20^, before proceeding to co-express all four Es in a single *P. pastoris* host. In the current manuscript, we describe the co-expression and co-purification strategy adopted to obtain these tetravalent mVLPs (T-mVLPs) and present data on the comparison of their immunogenicity with that of two other tetravalent E-based VLP formulations: a physical mixture containing four monovalent VLPs (M-VLP mix) and a physical mixture of two bivalent mVLPs (B-mVLP mix).

## Results

### Multiple approaches to tetravalent DENV E VLP-based vaccines

**Figure 1:**
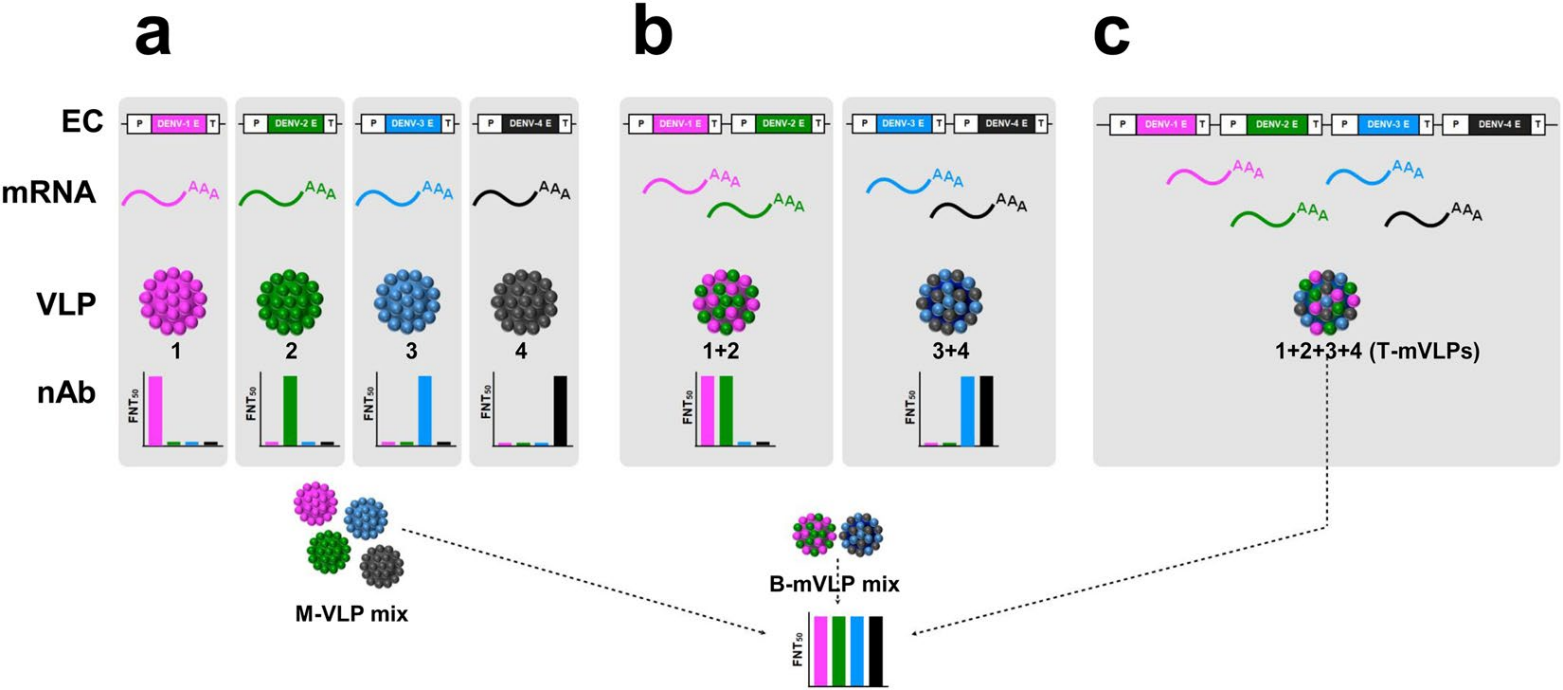
Schematic representation of different *P. pastoris*-expressed E-based tetravalent VLP dengue subunit vaccine candidates. The picture depicts three approaches to obtaining tetravalent dengue VLP subunit vaccine. (**a**) The DENV *E* genes are expressed separately to obtain four serotype-specific monovalent VLPs (1, 2, 3, 4), which elicit predominantly homotypic nAb responses^16–19^. Mixing these four together will result in a tetravalent VLP formulation (M-VLP mix). (**b**) The *E* genes of two DENV serotypes are co-expressed in a single *P. pastoris* host to obtain bivalent mVLPs, which elicit predominantly homotypic nAb responses specific to the two DENV serotypes they are derived from^20^. Mixing two different kinds of such bivalent mVLPs (1 + 2 and 3 + 4), together representing all four DENV serotypes, constitutes a second approach to a tetravalent VLP formulation (B-mVLP mix). (**c**) The *E* genes of all four DENV serotypes can be co-expressed in a single tetravalent *P. pastoris* host to obtain T-mVLPs containing all four E proteins (1 + 2 + 3 + 4). The T-mVLPs represent the third approach. Shown on top are the monovalent (**a**), bivalent (**b**), and tetravalent (**c**) expression cassettes (EC). P and T in each EC denote the *AOX1* promoter and terminator, respectively. The *E* genes of DENV-1, DENV-2, DENV-3 and DENV-4 are shown in magenta, green, blue and black, respectively. The same colour scheme is followed for the corresponding mRNAs, E proteins (in the VLPs) and the nAb responses (FNT_50_ histograms) to each of the four DENV serotypes. The VLPs, shown below the mRNAs, in panels a, b and c, are monovalent, bivalent and tetravalent, respectively. The DENV serotypes represented in each VLP species is shown by the Arabic numeral below the VLPs. The schematic FNT_50_ histogram at the bottom depicts the tetravalent nAb response predicted to be elicited by all three VLP candidates.

We assessed three *P. pastoris*-produced, prM-lacking, E-based tetravalent VLPs, shown schematically in Fig. 1. Mixing the four monovalent VLPs, referred to above, represents one tetravalent vaccine candidate (M-VLP mix). A physical mixture of two bivalent mVLPs (B-mVLP mix) represents a second tetravalent vaccine candidate. As we already had monovalent^16–19^ and bivalent^20^ *P. pastoris* clones at hand, we created a tetravalent *P. pastoris* clone, capable of co-expressing all four DENV E proteins, as a prelude to making head-to-head comparison of the immunogenicity of the resultant T-mVLPs with that of M-VLP mix and B-mVLP mix. The purpose of this comparative analysis was to ascertain if the T-mVLPs would retain the antigenic integrity and immunogenicity of their four monovalent precursors and serve as a single tetravalent dengue immunogen.

### Strategy to co-express E proteins of all four DENVs in *P. pastoris*

**Figure 2:**
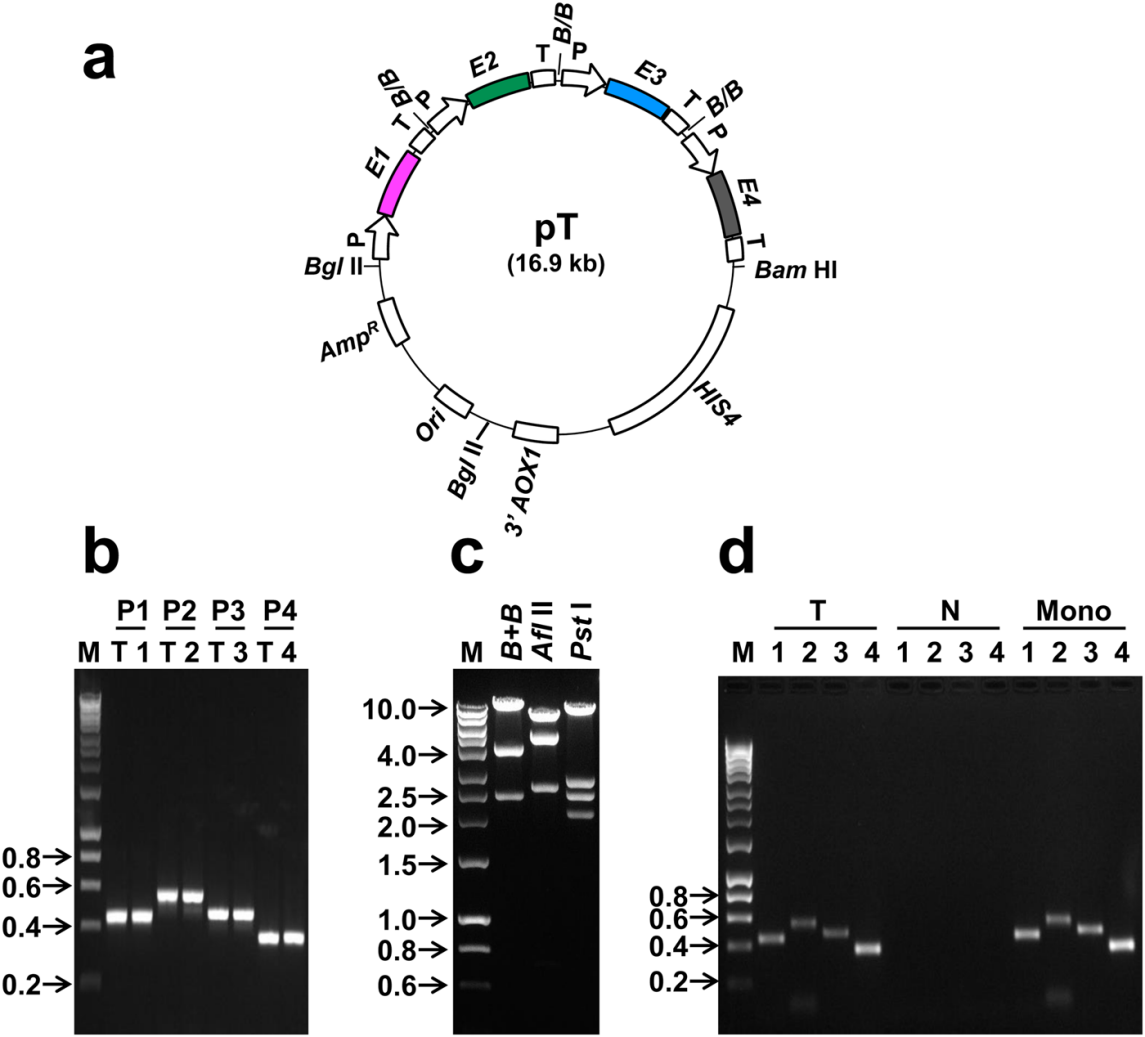
Strategy to create a single tetravalent *P. pastoris* clone. (**a**) Plasmid pT harboring four DENV *E* ECs. Each EC consists of the *AOX1* promoter (P), *E* gene of a single DENV serotype (*E1*: magenta, *E2*: green, *E3*: blue, *E4*: black) and the *AOX1* terminator (T). ‘B/B’ denotes the *Bam* HI/*Bgl* II fusion site created by the ligation of the 3′ end of one EC to the 5′ end of the next in the tandem array. The plasmid contains 3′ *AOX1* sequences and *HIS4* marker for integration and selection in *P. pastoris*, respectively, as well as bacterial sequences for replication (*Ori*) and ampicillin selection (*Amp*^*R*^) in *E. coli*. (**b**) Agarose gel analysis of PCR products obtained using plasmid pT (lanes ‘T’) as well as monovalent plasmids containing *E* gene of DENV-1 (lane 1), DENV-2 (lane 2), DENV-3 (lane 3) and DENV-4 (lane 4), as templates, in conjunction with primer pairs P1, P2, P3 and P4^20^, specific to genes *E1*, *E2*, *E3* and *E4*, respectively. (**c**) Agarose gel analysis of restriction digests of plasmid pT performed with *Bgl* II + *Bam* HI (B + B), *Afl* II and *Pst* I. (**d**) PCR analysis of *P. pastoris* genomic DNA. Genomic DNA from the tetravalent *P. pastoris* clone (T) and from untransformed *P. pastoris* host (N, negative control) were analysed by PCR for the presence of *E1* (lanes ‘1’), *E2* (lanes ‘2’), *E3* (lanes ‘3’) and *E4* (lanes ‘4’) genes, using the same gene-specific primer pairs as in panel ‘b’. Monovalent (Mono) *E1*, *E2*, *E3* and *E4* plasmid DNAs were analysed in parallel as positive controls (in ‘Mono’ lanes 1–4). Panels ‘b’ and ‘d’ represent full gels; the gel in panel ‘c’ is cropped to eliminate lanes in which unrelated samples were run (the full gel picture is included in supplementary file, page 5. For panels ‘b-d’, DNA size markers were analysed in lanes ‘M’. Their sizes (in kb) are shown to the left of each panel. Predicted sizes of PCR amplicons (panels ‘b’,‘d’), location of restriction sites and the expected restriction fragments (panel ‘c’) are indicated in Supplementary Fig. S2.

A single tetravalent plasmid vector, pT, capable of expressing all four *E* genes (Fig. 2a) was constructed as described (Supplementary Protocol S1 and Figs S1 and S2). The presence of all four *E* genes in pT was verified by PCR (Fig. 2b), using gene-specific primer pairs, and by restriction analyses (Fig. 2c). This was integrated into the *AOX*1 locus of *P. pastoris* GS115 (*his4*) and genomic DNA of several of the resulting clones was subjected to two rounds of PCR screening. Data on genomic PCR analysis of one typical clone is shown (Fig. 2d).

**Figure 3:**
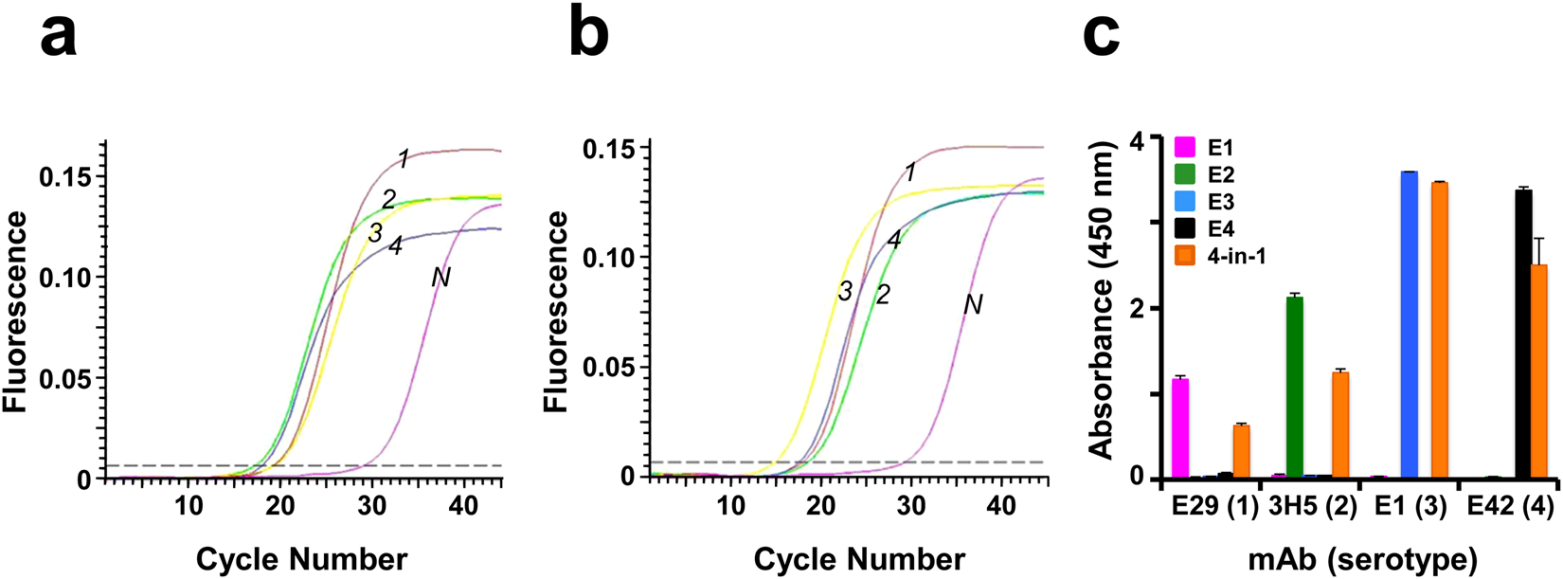
Analysis of co-expression of *E* mRNAs and proteins. (**a**) Real time analysis of DENV *E1*, *E2, E3* and *E4* mRNA co-expression in methanol-induced *P. pastoris* transformed with plasmid pT. Total RNA was isolated from the methanol-induced tetravalent clone of *P. pastoris*, transcribed to cDNA using reverse primers specific to *E1* (curve *1*), *E2* (curve *2*), *E3* (curve *3*) or *E4* (curve *4*) and subjected to qPCR. Total RNA from *P. pastoris* transformed with the empty expression vector pAO815 was subjected to RT-qPCR in parallel as a negative control (curve *N*). (**b**) Similar experiment as in panel ‘a’, except that mRNAs for real-time analysis were obtained from methanol-induced monovalent *P. pastoris* clones harboring *E1* (curve *1*), *E2* (curve *2*), *E3* (curve *3*) or *E4* (curve *4*) gene. (**c**) Analysis of co-expression of all four DENV E proteins with serotype-specific mAbs using indirect ELISA. Microtitre wells were coated either with methanol-induced *P. pastoris* lysate obtained from the tetravalent ‘4-in-1’ clone (orange bars) or purified E proteins obtained from the four different monovalent *P. pastoris* clones: E1 (magenta bar), E2 (green bar), E3 (blue bar) or E4 (black bar) proteins, and probed with mAbs specific to E proteins of DENV-1 [E29 (1)], DENV-2 [3H5 (2)], DENV-3 [E1 (3)] or DENV-4 [E42 (4)]. Bound mAbs were detected using secondary anti-mouse IgG-HRPO conjugate. Data shown are the mean of triplicates (error bars denote standard deviation, SD). Each experiment was performed at least twice independently with data from one typical experiment shown in each of the three panels.

Total RNA extracted from the methanol-induced tetravalent *P. pastoris* clone, was reverse-transcribed using primers complementary to each of the four *E* genes and followed by q-PCR (Supplementary Protocol S2), using primer pairs which were specific to each of the four *E* genes (Fig. 3a). Using identical conditions, we processed equivalent aliquots (normalised based on OD_600_) of methanol-induced *E1*-, *E*2*-*, *E*3- and *E*4-harboring monovalent *P. pastoris* clones for RNA extraction and RT-qPCR (Fig. 3b). The amplification profile and *C*_*t*_ value for any given *E* gene of the tetravalent clone was very similar and comparable to that of the corresponding monovalent clone, indicating that the tetravalent clone co-expresses all four *E* genes as efficiently as the monovalent clones. We analysed the insoluble pellet (P) fraction of the induced lysate of the tetravalent clone in small scale expression analysis using mAbs specific to each DENV serotype and found that it indeed co-expressed all four DENV E proteins (Fig. 3c). In this essentially qualitative analysis, the monovalent E VLPs used as references for the serotype-specificity of the mAbs were purified preparations. In contrast, the E proteins expressed by the T-mVLP clone were from a crude lysate (solubilised ‘P’ fraction), precluding meaningful quantitative comparison of ELISA reactivity data between the monovalent and tetravalent VLPs.

### E1, E2, E3 and E4 proteins co-purified under denaturing conditions co-assemble into VLPs

**Figure 4:**
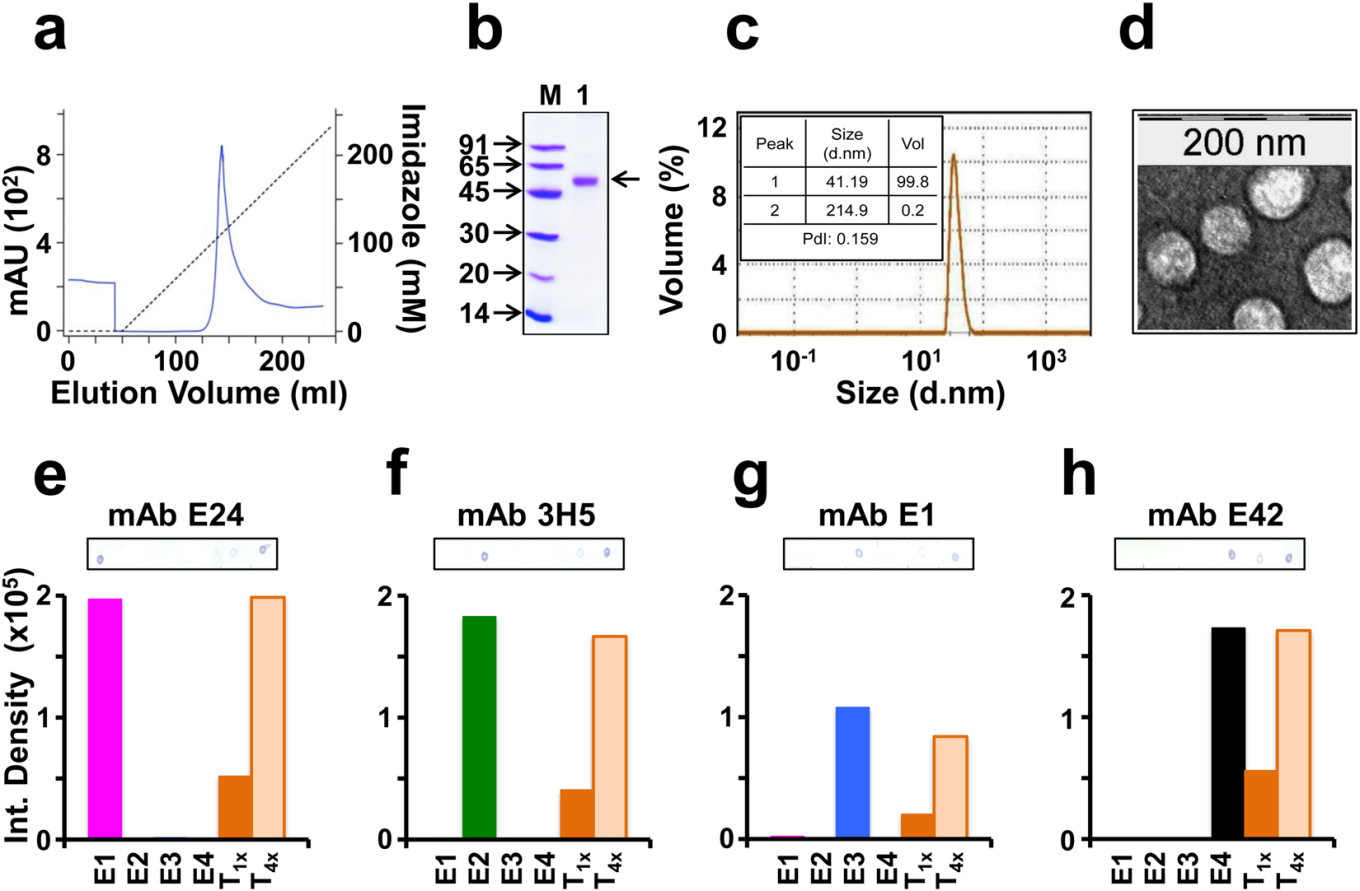
Co-purification and characterisation of co-expressed DENV E proteins. (**a**) Ni^2+^-NTA affinity chromatographic co-purification of E proteins co-expressed by the tetravalent *P. pastoris* clone. The solid blue curve represents the UV absorbance at 280 nm. The imidazole gradient is shown by the dashed black line. (**b**) SDS-PAGE analysis of the purified material (pooled peak fractions from panel ‘a’, lane 1) visualised by Coomassie blue staining. Protein size markers were run in lane ‘M’; their sizes (kDa) are shown on the left. This gel was run with just these two lanes and only these were scanned after staining. (**c**) DLS analysis of purified tetravalent mVLPs. The inset table shows the values of the DLS parameters. (**d**) EM picture of the VLPs after negative staining with uranyl acetate. The scale is shown at the top. (**e–h**) Dot blot assay to determine the relative proportion of E1, E2, E3 and E4 in T-mVLPs (details in Supplementary Protocol S3). Monovalent E VLPs corresponding to DENV serotypes 1 (E1), 2 (E2), 3 (E3) and 4 (E4) and T-mVLPs were spotted onto nitrocellulose strips and probed with anti-E mAbs specific to DENV-1 (mAb E24, panel ‘e’), DENV-2 (mAb 3H5, panel ‘f’), DENV-3 (mAb E1, panel ‘g’) and DENV-4 (mAb E42, panel ‘h’). T-mVLPs were spotted at two doses, the first (T_x_) equivalent to the same dose as the monovalent E VLPs and the second (T_4x_) at four times the first dose. The histograms indicate the intensity of the dots in the blot (shown above; raw blots are shown on page 5 of the supplementary file) scanned using ImageJ software. Reactivities of the mAbs to the monovalent E1, E2, E3 and E4 VLPs are indicated by magenta, green, blue and black bars, respectively, in the histograms; mAb reactivities to the lower (T_x_) and four-fold higher (T_4x_) doses of Tm-VLPs are indicated by the dark and light orange histogram bars, respectively. T-mVLP signal in the case of each serotype-specific mAb was equivalent to that of the corresponding monovalent E VLP when it was at 4x concentration indicating that it contains all four E proteins at 1:1:1:1 ratio.

All the four Es were co-purified using Ni^2+^-NTA affinity chromatography, starting from the insoluble P fraction, under denaturing conditions. A single sharp peak was eluted out of the chromatographic column at ~150 mM imidazole, as shown in Fig. 4a. SDS-PAGE analysis of an aliquot of the pooled peak fractions revealed a single protein band just above the 45 kDa marker (Fig. 4b), the predicted size of any of the four DENV E proteins. Purity was judged to be ~95% based on densitometric analysis. As the RT-qPCR analysis revealed that all four *E* mRNAs are co-expressed by the induced tetravalent clone, this major protein band in the pooled peak material must contain all four E proteins (see below). As all four E proteins are similar in size, this single band in the denaturing gel represents the co-migration of all four proteins. We have observed earlier that the affinity-purified *P. pastoris*-expressed monovalent E proteins self-assemble into discrete VLPs^16–19^. Further, we also observed that co-expression of two different types of DENV E proteins results in their co-assembly into B-mVLPs^20^. Thus, it is likely that co-expression of all four E proteins also could lead to their co-assembly into higher order structures. The pooled peak fractions were dialysed to remove urea and imidazole and subjected to dynamic light scattering (DLS) analysis (Fig. 4c). This showed that >99% of the dialysed material was comprised of ~40 nm particles. This was corroborated by visualisation of the VLPs using electron microscopy (EM) as shown in Fig. 4d. Taken together, these data demonstrate that the co-purified E proteins co-assemble into VLPs under native conditions. That these VLPs contain all four E proteins was confirmed based on their recognition by a panel of serotype-specific anti-E mAbs (see below).

### VLPs purified from the induced tetravalent clone contain E1, E2, E3 and E4

Next, we probed the purified VLPs using a panel of ten DENV serotype-specific anti-E mAbs in an indirect ELISA approach. This was essentially a qualitative assay using non-optimised amounts of antigens and mAbs. Microtitre wells were coated with these ‘tetravalent’ VLPs (1 µg/well) and then allowed to react with serotype-specific anti-E mAbs. In parallel, monovalent E VLPs^16–19^ were used (at 0.25 µg/well) as references to validate the serotype-specificity of the mAbs. The data are summarised in Table 1. That each of these mAbs is specific to only one DENV serotype is evidenced by its reactivity with the monovalent E VLPs of that particular serotype alone, with no discernible reactivity towards the remaining three DENV serotypes. All the anti-E mAbs manifested significant levels of reactivity with the VLPs obtained from the tetravalent *P. pastoris* clone, indicating that they are tetravalent mosaics (T-mVLPs). It is noteworthy that the relative magnitudes of the ELISA reactivity of the serotype-specific mAbs used for testing the crude lysates (Fig. 3c) were comparable to that observed with the purified T-mVLPs (Table 1). That a majority of these mAbs is EDIII-specific indicates that EDIIIs of all four DENV serotypes are displayed well on the surface of these T-mVLPs. This is consistent with our previous observation that *P. pastoris*-produced monovalent and bivalent E VLPs display EDIII on their surfaces^16–20^. On EDIII is a potent neutralising epitope called the lateral ridge (LR) epitope^25,36,38^. Binding sites for many mAbs, such as 3H5, 8A1, E29 and E40 map to *aa* residues that are either part of or adjacent to the EDIII-LR epitope. Collectively, the ELISA data support the conclusion that T-mVLPs preserve the antigenic identity and integrity of their monomeric precursors. A semi-quantitative dot-blot assay (Supplementary Protocol S3) using carefully optimised amounts of VLPs and serotype-specific DENV mAbs suggested that the four E proteins are present in T-mVLPs in approximately equal amounts (Fig. 4e–h).

**Table 1:** Analysis of ELISA reactivity of T-mVLPs with DENV serotype-specific mAbs.

mAb	Serotype specificity	Epitope Specificity	ELISA OD_450 nm_				
			E1 VLPs	E2 VLPs	E3 VLPs	E4 VLPs	T-mVLPs
E29	DENV-1	EDIII	1.87	0.03	0.09	0.14	1.08
3H5	DENV-2	EDIII (FG loop of LR)	0.03	2.12	0.02	0.02	1.25
8A1	DENV-3	EDIII (A strand, FG loop of LR)	0.02	0.01	0.91	0.01	0.85
E1		EDIII (C-C’ loop)	0.04	0.03	3.43	0.02	3.39
E3		EDI/II	0.13	0.03	2.90	0.02	2.04
E4			0.12	0.04	3.18	0.03	2.15
E29	DENV-4	EDIII (F and G strands)	0.04	0.04	0.03	2.80	2.23
E40		EDIII (A, F and G strands)	0.01	0.01	0.01	3.23	2.68
E42		E	0.03	0.02	0.02	2.10	2.70
E43			0.02	0.02	0.02	2.15	1.95

Indirect ELISA was performed using E1 VLPs, E2 VLPs, E3 VLPs, E4 VLPs or T-mVLPs as coating antigens. Bound VLPs were detected using DENV-1 mAb E29^26^, DENV-2 mAb 3H5^35^, DENV-3 mAbs E1, E3, E4^36^ and 8A1^37^, or DENV-4 mAbs E29, E42 and E43^39^. Mutational studies have identified specific *aa* residues recognising many of these mAbs. DENV-2 mAb 3H5 binds *aa* residues in FG loop of EDIII-LR^38^, DENV-3 mAb E1 binds G340 of C-C’ loop in EDIII^36^, DENV-4 mAb E29 binds to Y377 (F strand) and H390 (G strand) in the vicinity of EDIII-LR^39^ and DENV-4 mAb E40 binds K310 (A strand), D375, Y377 (F strand) and H390, F392 (G strand) in the vicinity of EDIII-LR^39^. Underlined ELISA OD_450 nm_ values indicate the serotype-specificity of the mAbs used to probe T-mVLPs. Data shown are from one of two separate experiments. Note: This assay was not optimised for quantitation of the relative proportion of the different E proteins in the T-mVLPs. Differences in ELISA reactivity of a given mAb between the T-mVLPs and the cognate monovalent E VLPs may reflect subtle differences in the accessibility of the EDIII epitopes in the tetravalent versus the monovalent VLPs. However, meaningful inter-serotypic comparison of reactivity between mAbs is precluded as each binds to distinct and unique epitope(s) of different serotypes, presumably with characteristic binding affinities.

As the indirect ELISA and the dot-blot assay used to detect Es of all four DENV serotypes do not differentiate between the monomeric and VLP forms of the E proteins, it is not possible to assess the proportion of free E monomers and the proportion of VLPs that may be composed of one, two, three or all four E proteins in the purified T-mVLP preparations. However, the relative proportions of the different putative kinds of VLPs in the T-mVLP preparation may not be quite relevant based on the induction of nAb titres to all four DENV serotypes (see below).

### T-mVLPs are highly immunogenic in BALB/c mice

**Figure 5:**
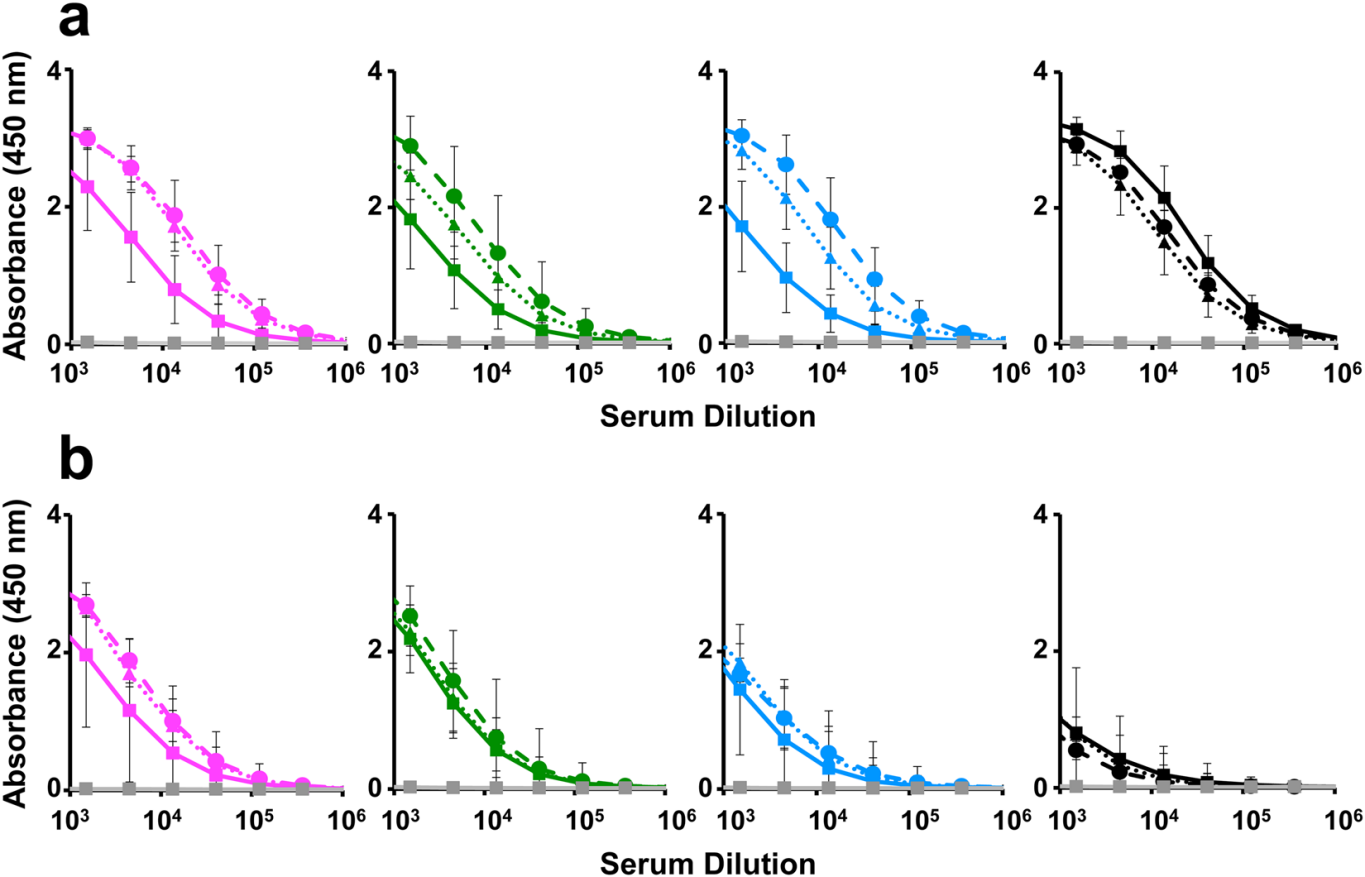
Evaluation of immunogenicity of E-based VLPs in BALB/c mice. Single immune sera from BALB/c mice (*n* = 6 per group), immunised with T-mVLPs (solid curves), B-mVLP mix (dashed curves) or M-VLP mix (dotted curves), all adsorbed on alhydrogel, collected 2 weeks after the last dose (days 0, 30 and 90), were analysed by indirect ELISA using all four DENV E (top four panels) or all four EDIII (as MBP fusions) proteins (bottom four panels), as coating antigens. In each of the panels, DENV serotypes 1, 2, 3 and 4, are indicated in magenta, green, blue and black, respectively. Mock-immunised (PBS on alhydrogel) BALB/c serum is represented in gray in all the eight panels. Data points represent geometric mean absorbance values (*n* = 6) with the error bars denoting SD. The ELISA response curves were essentially similar using pooled sera, analysed in parallel, from the three immunisation groups (Supplementary Fig. S3 and Table S1).

Next, we evaluated the immunogenicity of T-mVLPs in a head-to-head comparison with M-VLP mix and B-mVLP mix in BALB/c mice. Pooled (Supplementary Fig. S3) as well as single immune sera (Fig. 5a) were analysed by indirect ELISA using purified monovalent E VLPs as the capture antigens. Geometric mean titres (GMTs), for each VLP immunisation group, based on single sera data did not differ significantly from corresponding data obtained using pooled immune sera (Supplementary Table S1).

Immune sera from the T-mVLP group manifested very high titres (end point GMT: ~1 × 10^5^) of anti-E antibodies corresponding to DENV serotypes 1–3 (Fig. 5a). Anti-E GMTs in the immune sera from the M-VLP mix and B-mVLP mix groups were similar to each other but slightly higher (end point titres: ~3 × 10^5^). This difference in GMTs among the three immunisation groups vanished when the coating antigens were EDIII proteins instead (Fig. 5b). Interestingly, anti-E antibody GMTs against DENV serotype 4 were similar in all three immunogen groups. However, all three VLPs elicited relatively lower anti-EDIII antibody GMTs to DENV serotype 4 alone. Collectively, these data point to subtle differences between the immunogenicity of T-mVLPs on the one hand and the M-VLPs and B-mVLPs on the other. In terms of anti-EDIII antibody GMTs, the T-mVLP vaccine appears to elicit predominantly EDIII-focused antibodies, particularly to serotypes 1–3.

### Antibodies elicited by T-mVLPs tend to target EDIII and neutralise DENVs as effectively as those elicited by VLP mixtures

**Figure 6:**
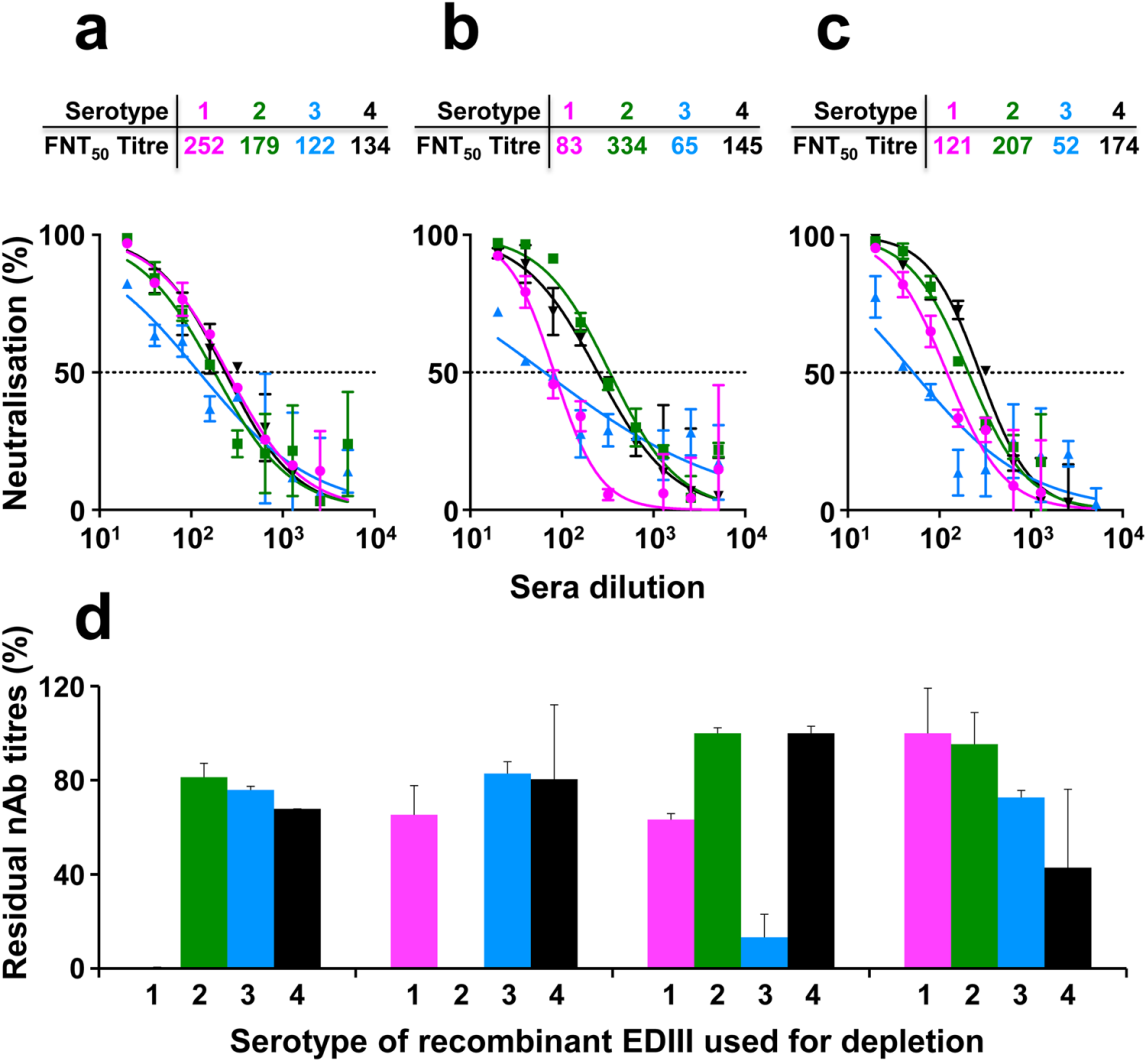
Evaluation of the induction of DENV-nAbs by E-based tetravalent VLPs in BALB/c mice. Virus-neutralising activity of pooled (*n* = 6) antisera from mice immunised with (**a**) ‘M-VLP mix’, (**b**) ‘B-mVLP mix’ and (**c**) ‘T-mVLPs’ against WHO reference strains of DENV-1 (magenta), DENV-2 (green), DENV-3 (blue) and DENV-4 (black) as a function of serum dilution, determined using the FACS-based assay. The dashed horizontal line denotes 50% neutralisation of DENV infectivity. Data points represent mean (*n* = 6) values; the error bars represent SD. Tables above the graphs indicate nAb titres (FNT_50_) calculated from the graphic data. Mann-Whitney test-derived *p* values for DENV-1 (*p* = 0.7962), DENV-2 (*p* = 0.6665), DENV-3 (*p* = 0.2973) and DENV-4 (*p* = 0.8633) did not reveal any statistically significant differences in nAb titres between ‘T-mVLP and ‘B-mVLP mix’ groups. Similarly FNT_50_ titres between ‘T-mVLP’ and ‘M-VLP mix’ groups for DENV-1 (*p* = 0.5457), DENV-2 (*p* = 0.8633), DENV-3 (*p* = 0.6048) and DENV-4 (*p* = 0.7962) did not vary significantly based on Mann-Whitney test for significance. (**d**) Pooled immune sera from the T-mVLP group was pre-depleted of EDIII-specific Abs on immobilised MBP-EDIII proteins corresponding to the four DENV serotypes (1, 2, 3 & 4) before determination of residual virus neutralising activity. Data are expressed as percent residual geometric mean nAb titres with respect to those of corresponding controls, mock-depleted on immobilised MBP, taken as 100%. Error bars denote SD. Mock-depletion did not significantly affect the nAb titres specific to the four DENV serotypes compared to un-depleted immune sera (Supplementary Fig. S4).

We performed a fluorescence activated cell sorting (FACS)-based assay^40^ to determine the nAb titres elicited by T-mVLPs against each of the four DENV serotypes, and compared these to corresponding nAb titres elicited by the monovalent and bivalent VLP mixes (Fig. 6). For this we used pooled sera as they are representative of individual sera, based on the comparative analysis of immunogenicity above (Fig. 5, Supplementary Table S1 and Fig. S3). The data revealed that all three antisera pools possessed the capacity to neutralise each of the four DENVs. A comparison of the FNT_50_ titres revealed that differences among the three immunisation groups were not statistically significant. This was true for each of the four DENVs. These data show that, in terms of nAb-inducing potential, the T-mVLPs are essentially equivalent to the monovalent and bivalent VLP mixes. In other words, one can achieve tetravalent virus-neutralising seroconversion using a single T-mVLP immunogen as efficiently as those achievable using tetravalent formulations comprised of multiple VLP immunogens. That these nAbs are predominantly EDIII-focused and serotype-specific has been demonstrated in previous work with monovalent^17–19^ and bivalent VLPs^20^. We found that pre-incubating the anti-T-mVLP antisera with recombinant EDIII-maltose-binding protein (MBP) fusion proteins corresponding to the four DENV serotypes resulted in large losses in homotypic, but not heterotypic, nAb titres (Fig. 6d). Control depletions done with MBP alone did not adversely affect nAb titres (Supplementary Fig. S4). The data suggest that nAbs elicited by T-mVLPs are predominantly EDIII-directed. In fact, we recently found that displaying EDIIIs of the four DENV serotypes on a VLP platform is an effective way to elicit potent tetravalent nAbs^41^. The induction of pan-DENV nAbs is indicative of potential protective efficacy of the VLP vaccines against all four DENV serotypes. Different studies have documented an inverse relationship between nAb titres and the extent of risk of severe disease^7,42,43^. Nevertheless, it is important to ascertain that nAbs do not possess inherent ADE potential.

### Antibodies elicited by T-mVLPs, B-mVLPs and M-VLPs do not promote ADE *in vivo*

**Figure 7:**
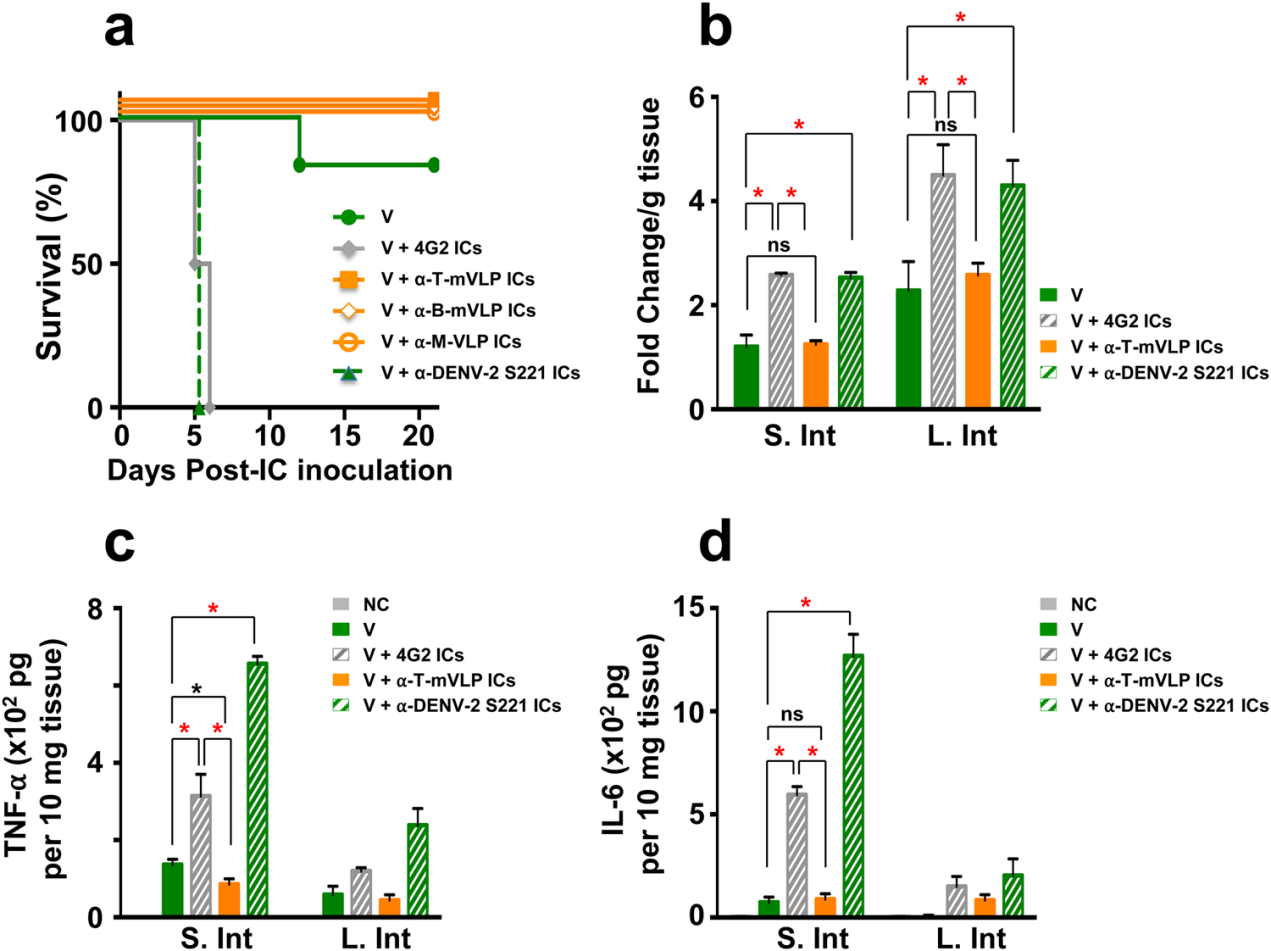
Determination of the ADE potential of antibodies elicited by DENV E-based tetravalent VLP vaccines in BALB/c mice. (**a**) Groups (*n* = 12) of AG129 mice were challenged with a sub-lethal dose of DENV-2 S221 (V) or fully-neutralised ICs generated *in vitro* by incubating the same sub-lethal dose with mAb 4G2 (V + 4G2 ICs), ‘anti-T-mVLP’ antiserum (V + α-T-mVLP ICs),’ anti-B-mVLP mix’ antiserum (V + α-B-mVLP ICs), ‘anti-M-VLP mix’ antiserum (V + α-M-VLP ICs) or anti-DENV-2 S221 antiserum (V + α-DENV-2 S221 ICs) and monitored for survival for a period of 3 weeks. Survival of the ‘V’ (*p* = 0.0009), V + α-T-mVLP ICs (*p* = 0.0009), V + α-B-mVLP ICs (*p* = 0.0009) and V + α-M-VLP ICs (*p* = 0.0009) groups were significantly higher compared to the survival in the ‘V + 4G2’ group, based on the Mantel-Cox test of significance. Mortality in the ‘V + α-DENV-2 S221 ICs’ group was not significantly different from that in the ‘V + 4G2’ group (*p* = 0.0555, Mantel-Cox test). Survival curves of multiple groups at 100% have been slightly displaced to avoid superimposition and make them visible. (**b**) A subset of AG129 mice (*n* = 3) from the ‘V’, ‘V + 4G2 ICs’, ‘V + α-T-mVLP ICs’ and the ‘V + α-DENV-2 S221 ICs’ were assessed for vascular leakage, in small (S. Int) and large intestines (L. Int), at 4 days post-IC inoculation by Evans blue staining assay. Data are depicted as fold-change in dye-staining with reference to a negative control (NC) group of AG129 mice which did not receive any treatment. A fold-change of 1 corresponds to basal vascular leakage equivalent to the NC group. (**c**) A second subset of AG129 mice (*n* = 3) from the same four groups listed in panel ‘b’ were euthanised 4 days after IC inoculation for the preparation of S. Int and L. Int tissue extracts followed by determination of TNF-α. (**d**) Determination of IL-6 in the same extracts prepared in panel ‘c’. In panels ‘c’ and ‘d’ NC refers to a negative control group of AG129 mice which did not receive any treatment. Statistically very significant and significant differences (un-paired *t* test) in panels ‘b-d’ are indicated by red and black asterisks, respectively; ns: not significant.

Though we had eliminated prM in our VLP vaccines, the likelihood that a subset of the anti-E polyclonal Ab repertoire may be cross-reactive and be potentially capable of ADE cannot be ruled out. Recent work has shown that ADE can be induced in DENV-susceptible AG129 mice, which lack interferon α/β and γ receptors, by injecting into them a non-lethal dose of the challenge virus DENV-2 S221 complexed to a cross-reactive antibody such as mAb 4G2^44^. We deployed this model system to examine if the antibodies elicited by the tetravalent E-based VLPs possessed ADE potential (Fig. 7a). A non-lethal dose of DENV-2 S221 pre-incubated with normal mouse serum (‘V’ group) did not cause any discernible mortality in AG129 mice. In striking contrast, an immune complex (IC) generated *in vitro* by incubating the same non-lethal dose of DENV-2 S221 with an amount of mAb 4G2 adequate to neutralise it fully (‘V + 4G2 ICs’ group) resulted in 100% mortality by post-inoculation day 6. It is to be noted that 4G2 is a cross-reactive mAb. Despite neutralising DENV-2 S221 fully, mAb 4G2 enhanced a non-lethal infection of AG129 mice into a lethal infection (*p* = 0.0009). This is consistent with previously reported work^20,41,44^. In striking similarity to this, we observed that fully-neutralised ICs generated by pre-incubating DENV-2 S221 with anti-DENV-2 S221 antiserum also resulted in rapid 100% mortality of AG129 mice (‘V + α-DENV-2 ICs’ group). This suggests that the anti-viral antiserum contains cross-reactive antibodies, presumably anti-prM antibodies among others, which possess significant ADE potential. Interestingly, when fully neutralised ICs were generated *in vitro* by incubating DENV-2 S221 with antisera from BALB/c mice immunised with B-mVLP mix (‘V + α-B-mVLP ICs’ group) or M-VLP mix (‘V + α-M-VLP ICs’ group), and inoculated into AG129 mice, there was no discernible mortality. This was true for DENV-2 S221 ICs made using antibodies elicited by T-mVLPs as well (‘V + α-T-mVLP ICs’ group). Survival of mice in this group did not differ significantly with respect to those in the ‘V’ group (*p* = 0.317). Clearly, the antibodies elicited by the E-based VLPs did not enhance the non-lethal DENV-2 S221 infection in AG129 mice. The induction of predominantly EDIII-focused serotype-specific nAbs, without enhancement potential, observed here is also reflected in recent work which demonstrated that cross-reactive, but not serotype-specific nAbs, mediate enhancement *in vivo* using the AG129 mouse model^20,44^. Recent data suggest that DENV anti-EDIII Abs do not enhance the related flavivirus, ZIKV, as well^45^.

### ICs generated using T-mVLP-induced antibodies do not cause vascular leakage or pro-inflammatory cytokine production in AG129 mice

Previous studies have shown that AG129 mice undergoing ADE in the IC inoculation experiment mentioned above, manifest evidence of increased vascular leakage as well as elevated production of the pro-inflammatory cytokines TNF-α and IL-6, particularly in the small intestinal tissue^44^. To corroborate the ADE data in Fig. 7a, we examined these three parameters in the ‘V + α-T-mVLP ICs’ group and compared these with ‘V’, ‘V + 4G2 ICs’ and ‘V + α-DENV-2 S221 ICs’ groups. We analysed vascular leakage in intestinal tissue using Evans blue staining (Fig. 7b) and cytokine production using ELISA (Fig. 7c,d), on day 4 post-IC challenge. Taking the level of Evans blue staining in the ‘V’ group, which did not manifest any significant mortality (Fig. 7a) as baseline, we did not observe any significant increase in the ‘V + α-T-mVLP ICs’ group, suggesting that anti-T-mVLP antibodies do not contribute to vascular leakage, either in the small intestines or in the large intestines. In contrast, however, Evans blue staining revealed a statistically significant increase in vascular leakage over basal levels, in the ‘V + 4G2 ICs’ and ‘V + α-DENV-2 S221 ICs’ groups, in both small and large intestines. Consistent with this, both these groups also showed significantly elevated levels of both TNF-α and IL-6, compared to the ‘V’ group. The levels of these two cytokines in the ‘V + α-T-mVLP ICs’ group were more or less comparable to those seen in the ‘V’ group. However, the cytokine elevation was more prominent in the small intestines of animals manifesting ADE. Strikingly, this was more pronounced in the ‘V + α-DENV-2 S221 ICs’ group suggesting that the antiviral antiserum has more potent ADE capacity. Interestingly, we observed that the reduction in these pro-inflammatory cytokine production seen at day 4 post IC-inoculation in the ‘V + α-T-mVLP ICs’ group persisted even at day 21 (Supplementary Fig. S5). Collectively, the vascular permeability and cytokine data strengthen the conclusion that antibodies elicited by T-mVLPs do not enhance a sub-lethal DENV-2 S221 infection in AG129 mice.

## Discussion

The existence of four DENV serotypes, the induction of lifetime homotypic, but not heterotypic immunity and the association of secondary infection with severe disease due to cross-reactive, non-neutralising antibodies from primary infection, collectively mandate that a preventive dengue vaccine must be tetravalent. However, the precise immune correlates of protection are yet to be elucidated^46^. Though nAbs are regarded as mediators of protection against flaviviral infections, in the case of DENVs, it appears that both humoral^43^ as well as cellular^47^ immunity may contribute to protection. Moreover, it is also recognised that incomplete or partial immunisation could carry the risk of serious dengue in the future^48^.

We are interested in non-LAV vaccines as they would not be faced with the issue of viral interference, which can compromise the induction of a balanced immune response, and they can be designed to eliminate viral antigens, such as prM, implicated in the induction of DENV infection-enhancing antibodies^27–29^. In this work, we have focused on VLP-based vaccines as these are highly immunogenic, capable of not only eliciting B cell responses, but can also through cross-presentation to the MHC-I pathway, elicit T cell responses as well^49^. However, there are limitations as well to VLP vaccines. For example, the duration of protection may decrease over time necessitating repeat immunisation(s).

Based on prior observations that all *P. pastoris*-expressed DENV E proteins possess inherent self-assembling potential^16–19^, and that co-expression of the E proteins of two different DENV serotypes leads to their co-assembly into bivalent mVLPs^20^, we have now explored the feasibility of co-expressing all four DENV E proteins in a single *P. pastoris* host to obtain a single tetravalent immunogen to assess its utility as a possible dengue vaccine candidate. To this end, we compared T-mVLPs to two different tetravalent physical mixtures of E-based bivalent (B-mVLP mix) and monovalent (M-VLP mix) VLPs. The objective was to find if the T-mVLPs would be equivalent, in terms of vaccine potential, to the tetravalent physical mixtures of either bivalent or monovalent VLPs. This would then obviate the need to express and purify more than one kind of VLP and help in cost-reduction.

In order to assess if the incorporation of the E proteins into T-mVLPs influenced their individual immunogenic potencies, we carried out a head-to-head comparison of these with a physical mixture of monovalent and bivalent VLPs. The T-mVLPs were highly immunogenic, eliciting antibodies that recognised all four DENV serotypes, using recombinant E proteins and EDIII proteins as coating antigens in an indirect ELISA format. Antibodies elicited by the three different VLP immunogens manifested some serotype-specific differences in ELISA reactivity towards recombinant E proteins. This may point to subtle differences in the disposition of the E monomers/epitopes in the three different VLPs. Interestingly, the antibody titres for the three different VLP immunogens were quite comparable to each other using EDIII as the capture antigen for DENV serotypes 1–3. The reason for the relatively lower immunogenicity of the EDIII moiety of the E4 protein is not clear. Taken together with earlier findings, our data suggest that *P. pastoris*-derived E VLPs, regardless of whether they are monovalent, bivalent or tetravalent, all tend to display the EDIII moiety to the immune system. Overall, the antigenic integrity and accessibility of EDIIIs of different E protein components in T-mVLPs are maintained intact as in the monovalent VLPs. Consistent with this, we found that T-mVLPs elicited nAbs which were effective against all four DENVs. In terms of nAb titres, T-mVLPs were more or less comparable to the M-VLP and B-mVLP mixes. This could mean that the pan-DENV nAbs elicited by the VLP vaccines comprise four subsets of nAbs, with each subset specific to one DENV serotypes. Our data revealed that pre-depletion of anti-T-mVLP antisera with recombinant EDIII of a given serotype resulted in a fairly selective abrogation of nAb titres corresponding to the cognate serotype. Given that DENV EDIIIs contain predominantly serotype-specific epitopes, we may conclude that our T-mVLP vaccines elicit nAbs to all four DENV serotypes. However, this does not necessarily rule out the induction of broadly-specific nAbs, effective against all DENV serotypes.

The corollary one may deduce from the ADE hypothesis is that enhancement of any DENV serotype by heterotypic Abs would be precluded in the presence of adequate levels of homotypic nAbs to all four DENV serotypes. This leads to the suggestion that antibodies elicited by the T-mVLPs (as well as B-mVLPs and M-VLPs mixes) would not enhance DENV infection. That the E-based VLPs elicit homotypic nAbs, referred to above, is an important finding, as recent work has indicated that serotype-specific nAbs do not manifest ADE in an *in vivo* mouse model^44^. We corroborated this conclusion experimentally by generating ICs of a non-lethal dose of the challenge virus, DENV-2 S221, with antibodies elicited by T-mVLPs (as well as B-mVLPs and M-VLPs) in BALB/c mice, and inoculating them into AG129 mice. The anti-T-mVLP antibodies (as also the anti-B-mVLP and anti-M-VLP antibodies) did not enhance the non-lethal infection into a lethal infection whereas the cross-reactive mAb 4G2 (or DENV-2 S221 antiserum) vigorously mediated ADE. Interestingly, in a recent study we observed that antibodies elicited by B-mVLPs consisting of E3 and E4 proteins, but not E1 and E2 proteins, enhanced DENV-2 S221 infection in a similar IC inoculation experiment in AG129 mice^20^. Taken together, these data are consistent with the corollary deduced from the ADE hypothesis.

AG129 mice manifesting ADE upon challenge with DENV-2 S221 + mAb 4G2 ICs display an intestinal pathology marked by capillary leakage and the production of pro-inflammatory cytokines, TNF-α and IL-6^44^. Consistent with this, we observed capillary leakage, as well as elevated TNF-α and IL-6 levels in the intestines of mice in the ‘V + mAb 4G2 ICs’ and ‘V + α-DENV-2 S221 ICs’ groups. In contrast, DENV-2 S221 ICs generated with antisera from all three groups of E-based tetravalent VLPs did not manifest capillary leakage or elevation in pro-inflammatory cytokine production in the intestinal tissue. Our data strongly suggest that antibodies induced by all three kinds of tetravalent E-based VLPs do not cause ADE *in vivo*. Given the limitations of the AG129 mouse on which this ADE model is based, these interpretations need to be tempered with caution. Further, we have explored ADE of DENV-2 infection alone here. Obviously, these studies need to be extended to the other three DENV serotypes when adequately characterised challenge strains become available.

Collectively, our data provide proof-of-concept that incorporation of all four E proteins into B-mVLPs^20^ and T-mVLPs (this study) does not compromise the immunogenic potential of the individual monomers. The T-mVLP in particular can contribute to further cost reduction as it entails a single expression and purification. Taken together, our data warrant further exploration of the *P. pastoris*-based T-mVLP as an alternate next generation dengue vaccine candidate.

## Materials and Methods

### Ethics statement

Animal experiments were carried out in accordance with the Committee for the Purpose of Control and Supervision of Experimental Animals (CPCSEA) guidelines of Government of India. BALB/c (ICGEB/IAEC/08/2016/RGP-14) and AG129 (ICGEB/IAEC/08/2017/01/RGP-16) mouse experiments were approved by the Institutional Animal Ethics Committee (IAEC) of ICGEB, New Delhi.

### Materials

Synthetic *E*1 (JX292264), *E*2 (JX292265)*, E*3 (JX292266) *and E*4 (JX292267) genes and the corresponding proteins, E1, E2, E3 and E4, have been described before^16–19^. The yeast host (*P. pastoris* strain GS115) and vector (pAO815) were from Invitrogen (Thermo Fisher Scientific), Carlsbad, USA. C6/36 and Vero cells were obtained from ATCC, VA, USA. The WHO DENV strains [DENV-1 (WP 74), DENV-2 (S16803), DENV-3 (CH53489) and DENV-4 (TVP-360)] have been described earlier^40^. DENVs were propagated in C6/36 cells to prepare stocks which were titrated on Vero cells using a FACS assay. Titres of the DENV stocks were expressed as FACS infectious units (FIU)/ml, as described^50^. EDIII-MBP fusion protein-expressing *E. coli* clones were kind gifts from Dr. Aravinda de Silva, University of North Carolina, USA. Dengue mAbs^26,35–39^ and PCR primers^20^ have been described before.

### Generation of a *P. pastoris* clone harboring *E1, E2, E3 and E4* genes

The tetravalent E expression plasmid, pT, was created starting from the bivalent expression plasmids, pDENV-E1E2_bv_ and pDENV-E3E4_bv_^20^, as described (Supplementary Protocol S1). Plasmid pT was integrated into the alcohol oxidase 1 (*AOX1*) locus of *P. pastoris* GS115 (*his4*) and resulting transformants were selected on minimal plates lacking histidine and analyzed further for slow methanol utilisation (*mut*^S^) phenotype. Genomic DNA of several His^+^/*Mut*^S^ clones was analysed for integration of the four different *E* genes by PCR with *E1*-, *E2*-, *E3*- and *E4*-gene specific primer pairs as described^20^.

### Analysis of co-expression of *E1*, *E2, E3 and E4*

Lysates were prepared from methanol-induced cells of the tetravalent *P. pastoris* clone and total RNA extracted as reported earlier for the bivalent clones^20^. The RNAs were reverse transcribed and subjected to q-PCR as described (Supplementary Protocol S2) using previously described primers^20^. In parallel, control reactions were performed wherein total RNA was extracted from equivalent number of methanol-induced cells from *E1*-, *E2*-, *E3*- and *E4*-gene carrying monovalent *P. pastoris* clones^16–19^, and subjected to q-PCR using identical experimental conditions.

### Methanol-induction

Cultures of the tetravalent clone were grown to log phase in yeast extract-peptone-dextrose medium and (a) induced for 24 hours using a range of methanol concentrations or (b) induced with 1% methanol for different time durations. Protein expression in total extracts was monitored using mAb 2412^51^ which recognises E1, E2 and E3 proteins. Based on these studies, methanol at 1.25% (v/v), added at 12-hourly intervals, for 72 hours resulted in maximal induction.

### Localisation of protein expression

Methanol-induced cells (equivalent to 100 OD_600_) of the tetravalent clone were lysed with glass beads (450 microns) in 0.5 ml of 1 × PBS and separated into supernatant (S) and membrane-enriched pellet (P) fractions. The latter was solubilised in 8 M urea and clarified by centrifugation. Proteins in the ‘S’ and urea-solubilised ‘P’ fraction were analysed by indirect ELISA using mAbE29^26^, mAb3H5^35^, mAbE1^36^ and mAbE42^39^, to detect the recombinant proteins E1, E2, E3 and E4, respectively. This showed that, similar to the situation with the monovalent clones, the proteins expressed by the tetravalent clone were also exclusively associated with the ‘P’ fraction.

### Co-purification of the E proteins expressed by the tetravalent clone

The E proteins co-expressed by the tetravalent *P. pastoris* clone were co-purified from the ‘P’ fraction of methanol-induced cell lysate, under denaturing conditions, by Ni^2+^-NTA affinity chromatography as reported earlier^16^. Purified proteins were analysed by SDS-PAGE followed by Coomassie blue staining. Purity was assessed using ImageJ software developed by NIH (https://imagej.nih.gov/ij/).

### Characterisation of the co-purified E proteins

Assembly of the co-purified proteins (at 200 µg/ml in 20 mM Tris-HCl/50 mM NaCl, pH 8.5) into higher order entities was assessed by DLS using Zetasizer Nano ZS90 (Malvern Instruments, Malvern, UK). VLP formation was visualised using a Tecnai electron microscope after 1% uranyl acetate staining of the purified material applied onto carbon-coated grids.

Integrity of the epitopes specific to the E proteins of all four DENV serotypes in the co-purified preparation was evaluated by indirect ELISA. For this, the purified E VLPs were used to coat ELISA plate microtitre wells, followed by determination of their reactivity towards a panel of ten mAbs (listed in Table 1) specific to the E proteins of each of the four DENV serotypes. Bound mAbs were revealed using anti-mouse IgG-HRPO conjugate/TMB substrate. This assay was purely a qualitative one designed to detect the different E proteins in T-mVLP preparations. After empirical optimisation of VLP and mAb amounts to obtain a linear dose-response, a semi-quantitative dot-blot assay was developed to assess the relative proportions of the four E proteins in purified T-mVLP preparation (Supplementary Protocol S3).

### Antigen formulation and immunisation

Tetravalent VLP formulations were prepared for testing in 3 groups of mice: (i) ‘T-mVLP’ group; (ii) ‘B-mVLP’ group; and (iii) ‘M-VLP’ group. In each case a single dose contained 80 µg VLP antigen, incubated with 500 µg alhydrogel for 36 hours at 4 °C (on a rocking platform), clarified by centrifugation (5,000 rpm, 5 minutes) and re-suspended in 100 µl sterile 1 × PBS. The antigen comprised 80 µg ‘T-mVLP’ (first group), 40 µg each of the two bivalent VLPs in the ‘B-mVLP’ group or 20 µg each of the four monovalent VLPs in the ‘M-VLP’ group. Mock formulation contained 500 µg alhydrogel/100 µl sterile 1 × PBS per dose. Groups of BALB/c mice (4–6 week old, *n* = 6) were immunised intramuscularly on days 0, 30 and 90 (80 µg VLPs/100 µl/dose). The dose of 20 µg/dose/serotype and immunisation schedule were based on prior work^16^. Sera were collected on day 105 *via* the retro-orbital route.

### Seroanalysis

Total antibody titres in the three groups of antisera (single and pooled) were determined by indirect ELISA using two sets of four ‘in-house’-purified coating antigens each: (i) *P. pastoris*-expressed, monovalent E1, E2, E3 and E4 proteins and (ii) *E. coli-*expressed recombinant MBP-EDIII fusion proteins corresponding to the four serotypes, MBP-EDIII-1, MBP-EDIII-2, MBP-EDIII-3 and MBP-EDIII-4. Bound antibodies were detected using anti-mouse IgG-HRPO conjugate/TMB substrate. To calculate end-point titres, a cut-off was generated by adding 2 × SD to the mean ELISA absorbance of the mock-immunised sera (*n* = 3) tested at the lowest dilution.

DENV-nAb titres, specific to a WHO panel of DENVs corresponding to the four serotypes, were determined using a FACS-based virus neutralisation assay as described before^40^. The serum dilution resulting in a 50% reduction in virus infectivity, compared to the cognate virus infectivity in the absence of any immune serum, is reported as the FACS neutralisation titre (FNT_50_). In some experiments, EDIII-specific antibodies were depleted from immune sera with immobilised MBP-EDIII proteins as described^20^, prior to nAb titre determination.

### Evaluation of ADE potential of antisera

Aliquots of DENV-2 S221 (2 × 10^4^ FIU) were separately incubated with mAb 4G2 (10 μg) or antiserum (5 μl neat) from each of the three immunisation groups, referred to above, for 1 hour on ice in a total volume of 50 μl (1 dose) to generate fully neutralised ICs. Each IC was prepared in excess of that required for 12 doses. The excess amount was tested by FACS assay to ensure that the virus in the IC was fully neutralised before injecting intravenously into groups (*n* = 12/group) of AG129 mice. A fifth group of AG129 mice received infectious DENV-2 S221 alone (2 × 10^4^ FIU/mouse). Six mice from each group were set aside for determination of vascular leakage and cytokine production (see below). The rest were monitored for survival for up to 3 weeks, and the data used to generate Kaplan-Meier survival curves.

To assess vascular leakage, 3 mice from each IC group were injected intravenously with 1% Evans blue solution in 1 × PBS (0.1 ml/mouse) on day 4 post-IC inoculation. The mice were euthanised 2 hours later. After thorough perfusion (with 1 × PBS), and removal of all visible luminal contents, equal weights of small and large intestines were collected into formamide (70 mg tissue in 500 µl) and extracted for 24 hours at 52 °C. Evans blue content in the extracts was determined by absorbance at 620 nm against formamide blank.

For cytokine analysis, a 2^nd^ set of 3 mice from each IC group above were euthanised on day 4 post-IC inoculation followed by perfusion and collection of small and large intestinal tissues^20^. These were used for total extract preparation using a Polytron homogeniser. The extracts were centrifuged and the supernatants used for the determination of TNF-α and IL-6, against biotinylated murine TNF-α and IL-6 standards, respectively, using commercial ELISA kits (Invitrogen; TNF-α: cat# KMC3011; IL-6: cat# KMC0061).

### Statistical analyses

GraphPad Prism software (version 7a) was used for statistical calculations. Probability (*p*) < 0.05 was considered statistically significant.

### Data availability

All data generated or analysed during this study are included in this published article (and its Supplementary Information file).

## Electronic supplementary material

Supplementary Information
