# Supplementary material for: Dengue envelope-based ‘four-in-one’ virus-like particles produced using Pichia pastoris induce enhancement-lacking, domain III-directed tetravalent neutralising antibodies in mice
Source: Sci Rep. 2018 Jun 5;8:8643. doi: 10.1038/s41598-018-26904-5 (PMC5988708; doi:10.1038/s41598-018-26904-5)
Supplement: Supplementary file 1 — Supplementary Information [file 41598_2018_26904_MOESM1_ESM.pdf]

# Dengue envelope-based 'four-in-one' virus-like particles produced using *Pichia pastoris* induce enhancement-lacking, domain III-directed tetravalent neutralizing antibodies in mice

**Ravi Kant Rajpoot[1¶], Rahul Shukla[1¶], Upasana Arora[1], Sathyamangalam Swaminathan[1]* and Navin Khanna[1,2,3]***

[1]*Recombinant Gene Products Group, Molecular Medicine Division, International Centre for Genetic Engineering & Biotechnology, New Delhi, INDIA*
[2]*Translational Health Science & Technology Institute, NCR Biotech Science Cluster, Faridabad, INDIA*
[3]*Department of Pediatrics, Division of Infectious Diseases, Emory University School of Medicine, Atlanta, GA, USA*

**Supplementary File**

## Protocol S1: Construction of plasmid pT for co-expression of all four DENV E proteins in *P. pastoris*

A single tetravalent plasmid vector capable of expressing all four *E* genes was constructed starting from two bivalent plasmids [20], one containing *E1* gene EC linked at its 3' end to the 5' end of *E2* EC (pDENV-E1E2$_{bv}$), and the other (pDENV-E3E4$_{bv}$) containing similarly head-to-tail linked *E3* and *E4* ECs (Fig. S1). The *E* gene of each EC encodes the carboxy terminal 34 *aa* residues of prM protein (signal peptide) of one DENV serotype followed by the amino-terminal 80% *aa* residues encoding the envelope ectodomain (E) of the same DENV serotype, followed by a 6x His tag, placed under the transcriptional control of the *AOX1* promoter on its 5' side and the *AOX1* transcriptional terminator on the 3' side. Further the linked bivalent EC unit in each of these two plasmids is flanked by 5' *Bgl* II and 3' *Bam* HI sites. To create a single tetravalent vector, the *E3+E4* ECs, retrieved as a *Bgl* II-*Bam* HI fragment from pDENV-E3E4$_{bv}$, was inserted into the unique *Bam* HI at the 3' end of *E2* EC in pDENV-E1E2$_{bv}$. The resulting tetravalent construct, pT, is designed to contain four independent ECs assembled in a tandem head-to-tail array. The presence of all four *E* genes in pT was verified by PCR (Fig. 2b; Fig. S2a), using gene-specific primer pairs, and by restriction analyses (Fig. 2c; Fig. S2b-d). A ~14.5 kb *Bgl* II fragment containing the four *E* gene ECs plus the *HIS4* marker was retrieved from this tetravalent construct and integrated into the *AOX1* locus of *P. pastoris* GS115 (*his4*) by electroporation. Transformants were selected on minimal plates lacking histidine and analysed further for acquisition of slow methanol utilisation ($mut^S$) phenotype, which indicates successful integration into the *AOX1* locus. Genomic DNA of several $His^+/Mut^S$ clones was subjected to two rounds of PCR screening. In the first round, clones were screened for the presence of the *E4* gene alone, followed by the second round to verify the integration of all four *E* genes, using *E1*-, *E2*-, *E3*- and *E4*-gene specific primer pairs, P1, P2, P3 and P4, respectively (Fig. 2d). One $His^+/Mut^S/E1^+/E2^+/E3^+/E4^+$ *P. pastoris* clone was identified for further work.

## Protocol S2: RT-qPCR

Total RNA (0.5 µg) from methanol-induced *P. pastoris* clones was reverse transcribed (20 µl reaction volume; incubation at 42°C for 1 hour) in four separate reactions using gene-specific primers RP1, RP2, RP3 and RP4, complementary to mRNAs corresponding to *E1*, *E2*, *E3* and *E4*, respectively, using iScript reverse transcriptase. After heat-inactivating the reverse transcriptase (85°C/5 minutes), each of the four cDNA products (5 µl after ten-fold dilution) was subjected to q-PCR in 25 µl reaction volume containing iQ SYBR green Supermix plus gene-specific primer pairs (RP1+FP1 for *E1* cDNA; RP2+FP2 for *E2* cDNA; RP3+FP3 for *E3* cDNA; and RP4+FP4 for *E4* cDNA) using a Bio-Rad MiniOpticon real-time PCR machine (cycling parameters: 94°C/3 min x1 cycle; 94°C/30 sec; 61°C/45 sec; 72°C/1 min x 42 cycles; melt curve analysis: 55°C-95°C). Primers have been described before [20].

## Protocol S3: Dot-blot analysis of T-mVLPs

A dot-blot assay was devised to evaluate the relative proportion of the four different E proteins in T-mVLPs as follows. The concentrations of each of the monovalent VLPs (E1, E2, E3 and E4) and the tetravalent VLPs (T-mVLP) spotted on nitrocellulose strips and the concentrations of the DENV serotype-specific mAbs for detecting these VLPs were optimised in multiple prior assays. After optimisation, a typical assay as shown in Fig. S3 was performed as follows. Nitrocellulose strips were spotted with purified E1, E2, E3, E4 and T-mVLPs. While the monovalent VLPs were spotted at a single dose, T-mVLPs were spotted at 2 doses, the first ($T_{1x}$) at the same dose level as the monovalent Es and the second ($T_{4x}$), at four-times the first dose. For probing with mAbs E24, 3H5 and E42 (all three mAbs at 1 µg/ml), E1, E2, E3, E4 and $T_{1x}$ spots contained 250 ng protein each with the $T_{4x}$ spot containing 1 µg protein. For probing with mAb E1 (at 15 ng/ml), E1, E2, E3, E4 and $T_{1x}$ spots contained 62.5 ng protein each with the $T_{4x}$ spot containing 250 ng protein. After spotting, the strips were blocked overnight at 4°C with 5% skim milk in 1x PBS. Blocked strips were incubated in primary antibody (the serotype-specific mAbs, in 2.5% skim milk in 1x PBS, at the indicated concentrations) for 1 hour at room temperature. The strips were then washed (5 times) with 0.1% Tween 20 in 1x PBS and incubated with goat anti-mouse IgG-HRPO (diluted 1:10,000 in 2.5% skim milk in 1x PBS) for 1 hour at room temperature. The strips were once again washed (5 times with 0.1% Tween 20 in 1x PBS) and developed with TMB substrate. Dried blots were scanned using NIH's ImageJ software to obtain integrated dot densities.

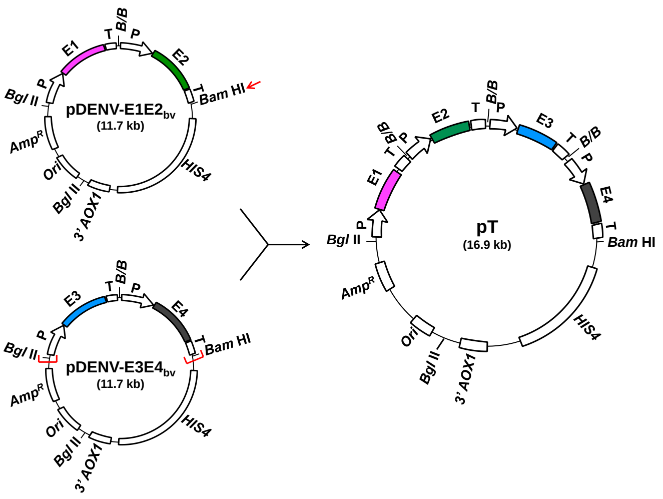


**Fig. S1: Schematic representation of the construction of plasmid pT.** The tetravalent plasmid pT was built from the two bivalent constructs pDENV-E1E2$_{bv}$ and pDENV-E3E4$_{bv}$ referred to above. Plasmid pT contains a head-to-tail tandem array of 4 ECs encoding DENV genes *E1* (magenta), *E2* (green), *E3* (blue) and *E4* (black). Each gene carries its own *AOX1* promoter (P) and terminator (T) on its 5' and 3' flanks, respectively. This tandem array was assembled by inserting the *Bgl* II-E3 EC-E4 EC-*Bam* HI fragment retrieved from pDENV-E3E4$_{bv}$ (indicated by the red square brackets) into the unique *Bam* HI site (indicated by the red arrow) of pDENV-E1E2$_{bv}$. A similar strategy was used to create the bivalent constructs starting from monovalent precursors [20]. 'B/B' denotes the *Bam* HI/*Bgl* II fusion site (resistant to both the restriction enzymes) created by the ligation of the 3' end of one EC to the 5' end of the next EC in the tandem array. All plasmids contain 3' *AOX1* sequences and *HIS4* marker for integration into *P. pastoris* GS115 (*his4*) as well as sequences for bacterial propagation (*Ori*) and selection (*Amp*$^{R}$).

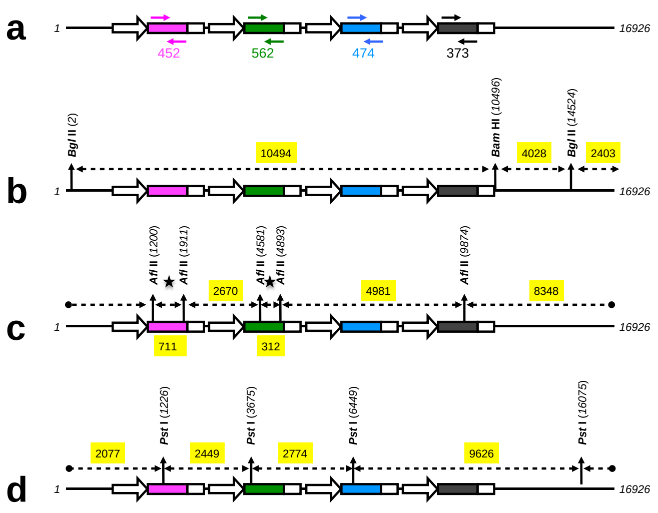


**Fig. S2: Linear representation of pT showing the locations of PCR primer-binding and restriction enzyme sites used for physical characterisation.** The linear pT genome is numbered arbitrarily starting at nucleotide (*nt*) 1 (close to the *Bgl* II site at the 5' side of *E1* EC of pT) to 16,926. The *E1*, *E2*, *E3* and *E4* genes are shown using the same colours as before. The rightward open arrow on the left and the open box on the right of each *E* gene denote P and T respectively of each EC. (a) The locations of the annealing sites for primer pairs P1, P2, P3 and P4 are shown relative to the positions of the *E1* (magenta box), *E2* (green box), *E3* (blue box) and *E4* (black box) genes, respectively. The forward (rightward) and reverse (leftward) primers are shown using short horizontal arrows coloured to match the corresponding *E* gene serotype. The numbers below the primer pairs denote the predicted amplicon size in base-pairs (*bp*). Panels 'b', 'c' and 'd' are line maps showing the location of *Bgl* II+*Bam* HI, *Afl* II and *Pst* I cleavage sites, respectively, on pT. The location of each of these restriction cleavage

sites is shown by the upward arrows with the corresponding *nt* number indicated in parentheses. The predicted restriction fragments are indicated by the double-headed dashed arrows (internal fragments), leftward dashed arrows (right terminal fragments) and rightward dashed arrows (left terminal fragments). Fragment sizes (in *bp*) are shown by the yellow highlighted numbers. The asterisks in panel 'c' denote *Afl* II fragments which are not easily discernible on the agarose gel due to low amount and small size.

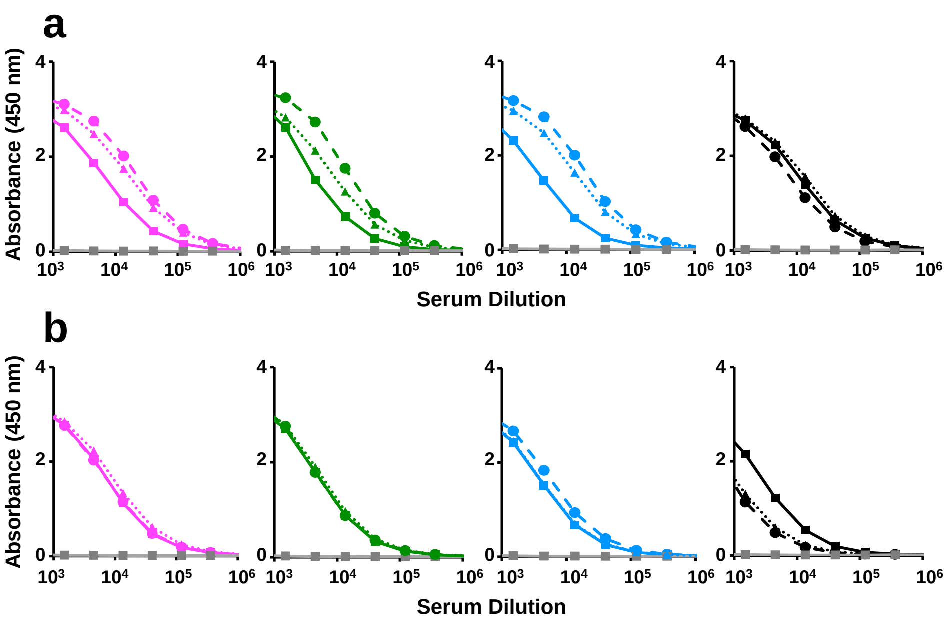


**Fig. S3: Evaluation of immunogenicity of E-based VLPs in BALB/c mice using pooled sera.** Pooled antisera from BALB/c mice (*n*=6 per group) immunised with T-mVLPs (solid curves), B-mVLP mix (dashed curves) or M-VLP mix (dotted curves), all adsorbed on alhydrogel, collected 2 weeks after the last dose (days 0, 30 and 90) were analysed by indirect ELISA using (a) all four DENV E (top four panels) or (b) all four EDIII (as MBP fusions) proteins (bottom four panels), as coating antigens. In each of the panels, DENV serotypes 1, 2, 3 and 4, are indicated in magenta, green, blue and black, respectively. Mock-immunised (PBS on alhydrogel) BALB/c serum is represented in gray in all the eight panels. Data points are mean absorbance values of three replicates of each serum pool.

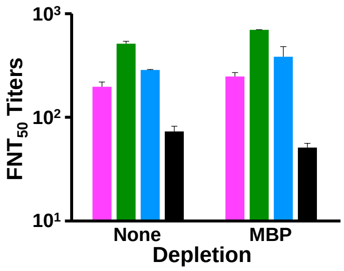


**Fig. S4: The fusion partner MBP does not affect virus neutralising activity of immune serum.** Pooled immune serum (*n*=6) from BALB/c mice immunised with T-mVLPs was assayed for DENV-neutralising activity either before (none) or after (MBP) passing through a column contaning immobilsed MBP. The data show geometric mean of nAb titers against DENV-1 (magenta), DENV-2 (green), DENV-3 (blue) and DENV-4 (black), with the error bars indicating SD.

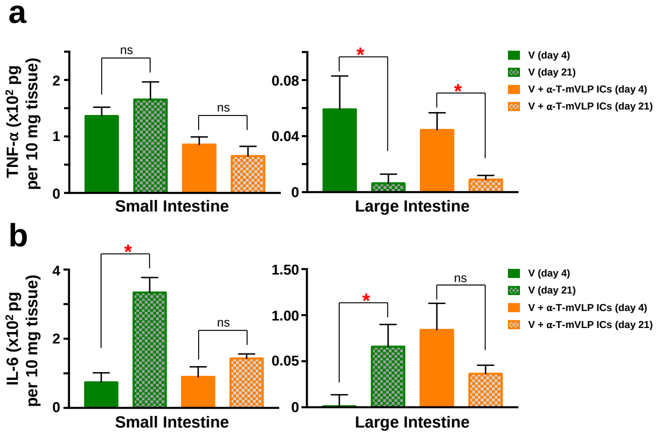


**Fig. S5: Comparison between pro-inflammatory cytokine levels in intestines of AG129 mice at days 4 and 21 post-IC inoculation.** The data shown are an extension of the experiment shown in Fig. 7 (panels c and d). At the conclusion of the experiment, on day 21 post-IC inoculation, mice (*n*=3) in the 'V' and 'V+α-TmVLP ICs' groups (these were the two surviving groups) were euthanised and intestinal extracts prepared as before for determination of TNF-α and IL-6. Paired *t* test was used to assess the significance of difference between day 4 versus day 21 cytokine levels. Statistically significant difference ($p<0.05$) is indicated by the red asterisks; ns: not significant.

**Table S1: Comparison of immunogenicity: Single versus pooled sera[a]**

| Antigen[b] | Mouse-1 | Mouse-2 | Mouse-3 | Mouse-4 | Mouse-5 | Mouse-6 |
|---|---|---|---|---|---|---|
| **Anti-T-mVLP antisera** | | | | | | |
| E1 | 0.7209 | 0.8785 | 0.7984 | 0.7209 | 0.2786 | 0.7209 |
| E2 | 0.1605 | 0.7984 | 0.8785 | 0.7984 | 0.7984 | 0.7209 |
| E3 | 0.2786 | 0.7209 | 0.9591 | 0.9591 | 0.2786 | 0.7209 |
| E4 | 0.1605 | 0.6454 | 0.5737 | 0.6454 | 0.2786 | 0.7209 |
| EDIII-1 | 0.7209 | 0.6454 | 0.3823 | 0.5737 | 0.0207 | 0.5054 |
| EDIII-2 | 0.3282 | 0.7209 | 0.4418 | >0.999 | 0.7984 | 0.7209 |
| EDIII-3 | 0.2786 | 0.7209 | 0.2786 | 0.7209 | 0.0830 | 0.7209 |
| EDIII-4 | 0.6454 | 0.7984 | 0.0070 | 0.7984 | 0.0830 | >0.999 |
| **Anti-B-mVLP antisera** | | | | | | |
| E1 | 0.7984 | 0.7984 | 0.7209 | 0.7209 | >0.999 | 0.7209 |
| E2 | 0.4418 | 0.7209 | 0.7209 | 0.7209 | 0.9591 | 0.2786 |
| E3 | 0.4418 | 0.7209 | 0.7209 | 0.7209 | 0.9591 | 0.5737 |
| E4 | 0.7984 | 0.7209 | 0.6454 | 0.7209 | 0.3823 | 0.7209 |
| EDIII-1 | 0.7209 | 0.6454 | 0.7209 | 0.7984 | 0.7209 | 0.7209 |
| EDIII-2 | 0.7209 | 0.7209 | 0.2786 | 0.7209 | 0.7209 | 0.7209 |
| EDIII-3 | 0.7984 | 0.5054 | 0.0207 | 0.7209 | 0.7209 | 0.7984 |
| EDIII-4 | 0.7209 | 0.5932 | 0.1049 | 0.7984 | 0.3823 | 0.0650 |
| **Anti-M-VLP antisera** | | | | | | |
| E1 | 0.8785 | 0.7209 | 0.7984 | 0.7209 | 0.9591 | 0.6454 |
| E2 | 0.8785 | 0.7984 | 0.7209 | 0.7428 | 0.3282 | 0.7209 |
| E3 | >0.999 | 0.7209 | 0.4418 | 0.7209 | 0.7984 | 0.7209 |
| E4 | 0.5737 | 0.7984 | 0.5737 | 0.7209 | 0.7984 | 0.8785 |
| EDIII-1 | 0.3823 | 0.2876 | 0.5737 | 0.8785 | 0.9591 | 0.8785 |
| EDIII-2 | 0.7209 | 0.7209 | 0.7209 | >0.999 | 0.7209 | 0.7209 |
| EDIII-3 | 0.2786 | 0.2786 | 0.7984 | 0.7209 | 0.7209 | 0.7984 |
| EDIII-4 | 0.7984 | 0.2786 | 0.7984 | 0.7209 | 0.2345 | >0.999 |

[a] Data shown are '*p*' values which were obtained from the indirect ELISA absorbance values using the Mann-Whitney test of significance (GraphPad Prism, v7a); '*p*' values (<0.05) indicating significant differences are shown in red fonts.
[b]Antigen denotes purified recombinant proteins used to coat the microtitre wells for indirect ELISA; E1, E2, E3 and E4 denote *P. pastoris*-expressed E VLPs corresponding to DENV serotypes 1, 2, 3 and 4, respectively; EDIII-1, EDIII-2, EDIII-3 and EDIII-4 denote *E. coli*-expressed EDIII-MBP fusion proteins in which the EDIII partner was from DENV serotypes 1, 2, 3 and 4, respectively.

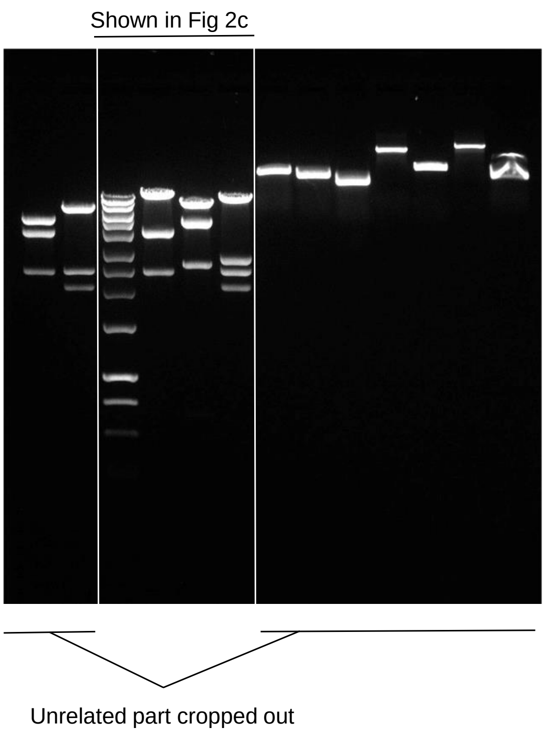
Shown in Fig 2c
Unrelated part cropped out

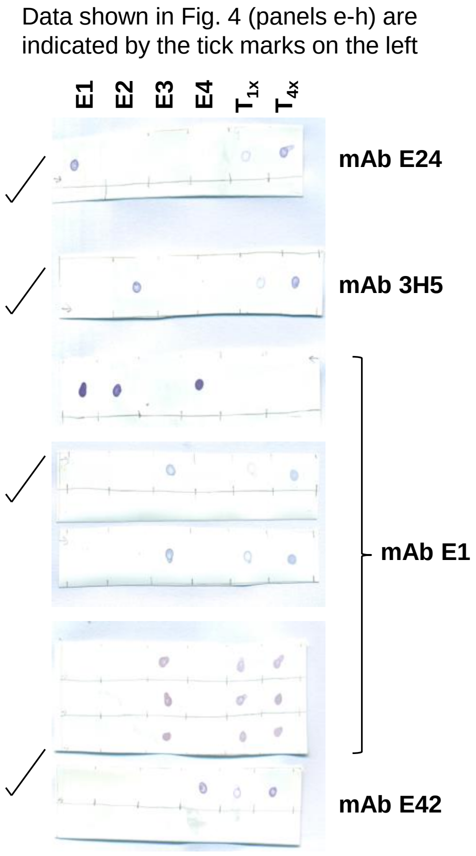
Data shown in Fig. 4 (panels e-h) are indicated by the tick marks on the left
E1
E2
E3
E4
T1x
T4x
mAb E24
mAb 3H5
mAb E1
mAb E42
